# Physiological and biochemical responses of two precious *Carpinus* species to high-concentration NO_2_ stress and their natural recovery

**DOI:** 10.1038/s41598-021-84702-y

**Published:** 2021-05-04

**Authors:** Qianqian Sheng, Min Song, Zunling Zhu, Fuliang Cao

**Affiliations:** 1 College of Landscape Architecture, Co-Innovation Center for Sustainable Forestry in Southern China, Nanjing Forestry University, Nanjing, 210037 China; 2 College of Landscape Architecture, Co-Innovation Center for Sustainable Forestry in Southern China, College of Art and Design, Nanjing Forestry University, Nanjing, 210037 China; 3 College of Forestry, Co-Innovation Center for Sustainable Forestry in Southern China, Nanjing Forestry of University, Nanjing, 210037 China

**Keywords:** Biodiversity, Ecophysiology, Forestry

## Abstract

*Carpinus betulus* and *Carpinus putoensis* are precious species in the world. Studies on the ecosystem function of the two species are rare. This study investigated the physiological and biochemical responses of *C. betulus* and *C. putoensis* to NO_2_ stress and their natural recovery. *C. betulus* and *C. putoensis* seedlings underwent fumigation with 12.0 mg/m^3^ NO_2_ for 0, 1, 6, 12, 24, 48, and 72 h, respectively. Then, the plants were allowed to recover at room temperature for 30 d. Physiological and biochemical changes in the leaves were compared between the two species. In terms of peroxidase (POD) activity, the damage response of *C. betulus* under NO_2_ stress appeared later than that of *C. putoensis*. The soluble protein content of *C. betulus* was noticeably higher than that of *C. putoensis*, and *C. betulus* exhibited more stable membrane lipoperoxidation. The tendency of the changes in nitrate reductase of *C. betulus* was less noticeable than that of *C. putoensis*. The variation amplitudes of N, K, Mg, Zn and Mn in the leaves of *C. putoensis* were greater than those of *C. betulus*. *C. putoensis* showed more sensitive metabolisms in response to NO_2_ stress compared with *C. betulus*. High-concentration NO_2_ caused damage to *C. betulus* and *C. putoensis* was reversible, and both species returned to normal growth via their own metabolism after 30-d recovery. The results of this study may provide useful reference data for quantitative assessment of the ecosystem function of *C. betulus* and *C. putoensis* and for their scientific application in urban greening.

## Introduction

Nitrogen dioxide (NO_2_) is a brownish red gas with a pungent odor. It has direct impact on atmospheric visibility and light absorption, and is a major atmospheric pollutant^1–4^. In the stratosphere, the NO_2_ content is relatively stable. However, in the troposphere, the content is not stable, which is mainly caused by industrial production and human activities, such as vehicle exhaust emission and thermal power station production^5,6^. Nowadays, atmospheric NO_2_ concentration has been considered as one important indicator in human pollution assessment^7^.

Landscape plants constitute an important part in constructing urban ecological environment. Plants do not only endow the city with seasonal changes, but also absorb and decompose harmful gases in the atmosphere. Furthermore, they cool, humidify and eliminate dusts and noise, thereby playing an important role in air purification and climate regulation. Under stress, a series of emergency reactions, including physiological changes and biochemical changes, may occur in plants to respond to the adverse situation. To date, scholars have conducted useful explorations of the emergency reactions of plants when exposed to NO_2_. Although some tree species exhibited seasonal differences in resistance against high-concentration NO_2_, all were observed with different degrees of damage between leaf veins: Some plants presented with brown damage spots and some showed yellow or white damage spots^8^. Under NO_2_ stress, biochemical changes of plants appear prior to the morphological and anatomical changes, which serve as early warning signals for modifications^9^. Malondialdehyde (MDA), an indicator for a variety of abiotic and biotic stresses that is caused by lipid peroxidation^10^, increases when white willow is confronted with high-concentration NO_2_^11^.

In the meantime, scholars have attempted to screen plants based on NO_2_ resistance capacity. Chen explored NO_2_ absorption and resistance of some landscape plants in Zhejiang, China, and reported that Theaceae and Lauraceae possessed relatively powerful NO_2_ resistance^12^. Li et al. carried out a study on the NO_2_ assimilation capacity of 70 woody plants. According to them, deciduous broad-leaved trees had the highest growth rate under high-concentration NO_2_ stress, among which *Robinia pseudoacacia*, *Sophora japonica*, *Pterocarya stenoptera*, and *Cerasus serrulata* exhibited the greatest recovery capacity^8^. Pan et al. observed the tolerance of 24 landscape plants to NO_2_ stress, and reported that the anti-stress capacities of *Cassia surattensis* and *Lagerstroemia indica* were the highest among the investigated plants^13^. Zhang et al. investigated the NO_2_ tolerance of 13 plant species in Yunnan Province, China, and found that *Acer paxii* had satisfactory resistance against NO_2_^14^. Liao et al. reported that *Camellia japonica* had the most powerful NO_2_ absorption ability among the landscape plants they investigated in Zhejiang, China^15^. The results of these studies indicate that broad-leaf species, particularly arbors and shrubs, own strong NO_2_ absorption capacity. Scholars have also explored treatment methods for plant damage caused by NO_2_. The addition of salicylic acids could relieve the damage caused by NO_2_ stress to *Triticum aestivum* to a certain degree^16^. Both the addition of salicylic acids and the symbiotic colonization of arbuscular mycorrhiza fungi could enhance the tolerance of *Avena nuda* to NO_2_; furthermore, these two treatments demonstrated a certain degree of synergistic effect^17^. Exogenous salicylic acids had positive regulatory effect on *Arabidopsis thaliana* under NO_2_ stress^18^. Pretreatment with H_2_O_2_ at a certain concentration improved the antioxidant capacity of *Brassica chinensis* and strengthened the tolerance of the plant to high-concentration NO_2_^19^. However, the results of all these studies suggest that plants can be adapted to adverse NO_2_ stress by adding chemical agents, and whether they can recover their normal metabolisms through self recovery remains to be explored.

*Carpinus betulus*, a broad-leaf deciduous tree originated from central Europe, is widely distributed in Europe and America nowadays. This plant species has a graceful shape with dense branches and leaves, which shows rich color changes according to seasons. These features make *C. betulus* an ideal species for landscaping. *Carpinus putoensis*, also a broad-leaf deciduous tree, is another precious species in the world. Currently, there is only one survived seed tree, which grows in Mount Putuo, Zhejiang Province, China. Therefore, *C. putoensis* has been listed as “a plant species at the critically endangered (CR) level” by the World Conservation Union (I-UCN). Over years, horticultural workers have carried out numerous research and practical works on the reproduction, breeding and landscape application of these two precious *Carpinus* species, and their application in landscaping has attracted increasing attention. However, research on the ecological function of these two *Carpinus* species, particularly their ability in air pollutant resistance, has not been reported. This reason restricts the application of the two species to a great extent. With regard to air pollutant resistance, particularly their resistance to NO_2_, studies are rare, except for the those conducted by Sheng and Zhu^20–22^, which devoted to the explorations of the changes in the photosysntheses and cellular anatomical structure of the two precious species under NO_2_ stress. The physiological responses of the plants under NO_2_ stress as well as the changes in mineral element contents in the leaves have not been reported, to the best of our knowledge.

Based on the aforementioned information, the current study investigated the physiological response (MDA, peroxidase (POD), soluble protein, nitrate reductase (NR) and nitrate nitrogen (NO_3_^−^N)) of and the mineral element content (N, P, K, Ca, Mg, Zn and Mn) changes in the leaves of *C. betulus* and *C. putoensis* under NO_2_ stress. Their self recovery through artificial cultivation at room temperature after stress removal was also explored. The results of this study might provide a theoretical foundation for plant selection for greening in urban areas contaminated by NO_2_.

## Results

### Morphological changes of the leaves

**Figure 1:**
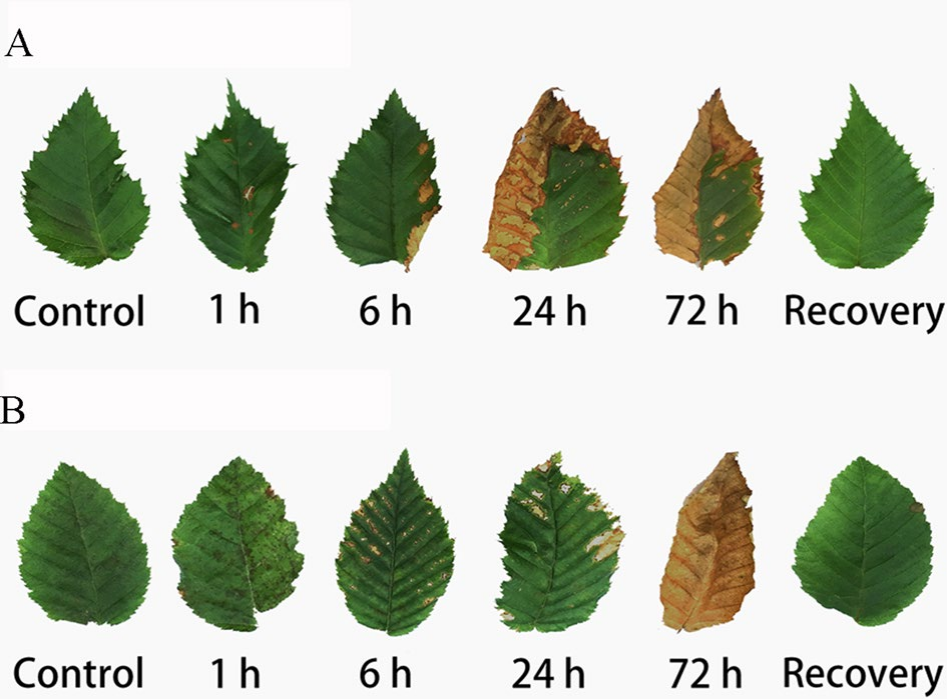
Leaf injury symptoms of *Carpinus betulus* (**A**) and *Carpinus putoensis* (**B**) under different NO_2_ exposure time and after recovery.

The influence of NO_2_ stress on the plants was first reflected by the morphological changes of the leaves (Fig. 1)^20,22^. Slight NO_2_ injury was manifested by slight green deficiency and light color. Moderate NO_2_ injury was manifested by irregular watery spots between leaf veins, which gradually developed into yellow necrotic spots followed by lesions at the leaf stalk and margins. When the exposure time extended to 72 h, the leaves turned yellow, and irreversible injury occurred, which led to leaf death. The damaged areas of the leaves of the two species at different time points of NO_2_ exposure are summarized in Table 1.

**Table 1:** The damaged areas (percentage) of the leaves of *Carpinus betulus* and *Carpinus putoensis* at different time points of NO_2_ stress.

Species	NO_2_ fumigation time				
	0 h	1 h	6 h (%)	24 h (%)	72 h (%)
*C. betulus*	0	0.52%	6.35%	49.43%	61.98%
*C. putoensis*	0	0	5.19%	9.97%	100.00%

### Changes in MDA content

**Figure 2:**
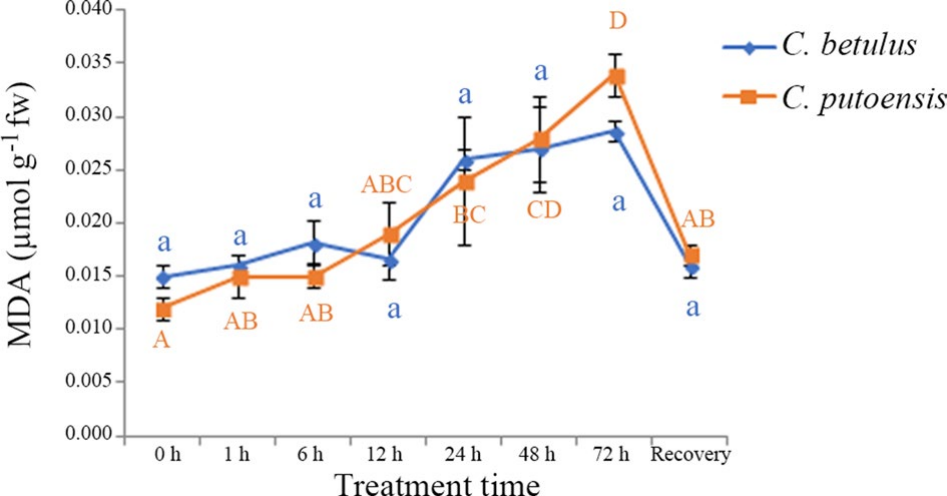
Changes in the MDA content of *C. betulus* and *C. putoensis* at different time points of NO_2_ stress and after self recovery. Letters or letter combinations containing the same letter indicate no significant difference between the corresponding NO_2_ exposure time points in the same plant species according to ANOVA or nonparametric Kruskal–Wallis ANOVA followed by Bonferroni tests. Capital letters for *C. putoensis* and lower letters for *C. betulus.*

The changes in the MDA content of *C. betulus* and *C. putoensis* at different time points of NO_2_ stress are shown in Fig. 2. With the prolongation of NO_2_ stress, the MDA content of *C. betulus* showed an increasing tendency with the variation range from 0.016 to 0.029 µmol g^−1^ fw. However, no significant differences were observed at different time points of NO_2_ exposure.

As NO_2_ fumigation time extended, the MDA content of *C. putoensis* also showed an increasing tendency, with the variation range from 0.015 to 0.034 µmol g^−1^ fw. Compared with the control group, a significant difference was observed in the MDA content from 24 h, which peaked at 72 h. In *C. putoensis*, although the recover group and the control group did not show a significant difference, the MDA content of the former lay between 0.015 (6 h) and 0.019 µmol g^−1^ fw (12 h), which suggests that the plant did not recovered completely from the stress damage.

Compared with *C. putoensis*, *C. betulus* exhibited a smaller variation amplitude in the MDA content under NO_2_ stress. The MDA content of *C. betulus* did not show noticeable changes at 1, 6, and 12 h, and it was till 24 h when a rapid increase occurred. These findings indicate a delayed injury response of *C. betulus*. In contrast, with the prolongation of NO_2_ stress, the MDA content of *C. putoensis* noticeably increased, which indicates an increase in the membrane lipid peroxidation activity of *C. putoensis* under NO_2_ stress.

### Changes in POD activity

**Figure 3:**
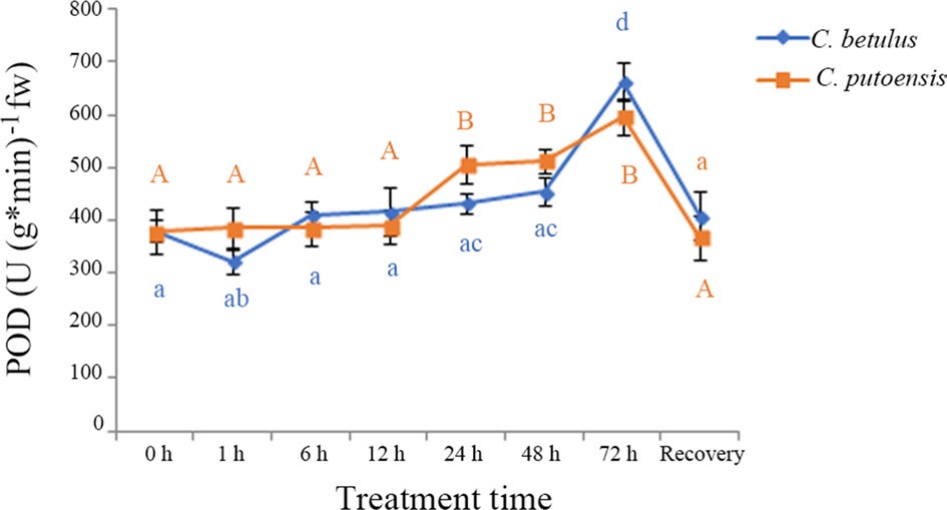
Changes in POD activity of *C. betulus* and *C. putoensis* at different time points of NO_2_ stress and after self recovery. Letters or letter combinations containing the same letter indicate no significant difference between the corresponding NO_2_ exposure time points in the same plant species according to ANOVA or nonparametric Kruskal–Wallis ANOVA followed by Bonferroni tests. Capital letters for *C. putoensis* and lower letters for *C. betulus.*

The changes in POD activity of *C. betulus* and *C. putoensis* at different time points of NO_2_ stress are shown in Fig. 3. With the prolongation of NO_2_ stress, the POD activity of *C. betulus* showed an increasing tendency, with a variation range from 323 to 663 U (g * min)^−1^ fw. After 30-d self recovery, the POD activity returned to 409 U (g * min)^−1^ fw, which was comparable to that of the control.

As NO_2_ fumigation time extended, the POD value of *C. putoensis* also showed an increasing tendency, with a variation range from 385 to 596 U (g * min)^−1^ fw. The recovery group did not show a significant difference compared with the control group.

In *C. betulus*, the POD activity value rapidly increased at 72 h of NO_2_ stress, which showed a significant difference compared with any other group (adjusted *p* < 0.05). In *C. putoensis*, however, a significantly increased POD value appeared from 24 h. These findings indicate that *C. putoensis* presented with injury response earlier than *C. betulus*.

### Changes in soluble protein content

**Figure 4:**
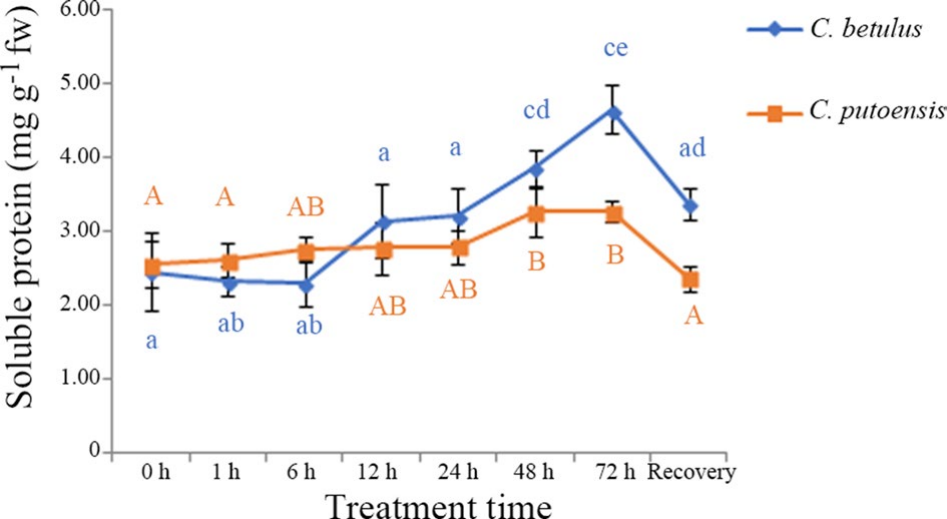
Changes in the soluble protein content of *C. betulus* and *C. putoensis* under NO_2_ stress at different time points and after self recovery. Letters or letter combinations containing the same letter indicate no significant difference between the corresponding NO_2_ exposure time points in the same plant species according to ANOVA or nonparametric Kruskal–Wallis ANOVA followed by Bonferroni tests. Capital letters for *C. putoensis* and lower letters for *C. betulus.*

The changes in the soluble protein content of *C. betulus* and *C. putoensis* under NO_2_ stress at different time points are shown in Fig. 4. Despite that the soluble protein content of *C. betulus* slightly decreased at 1 h and 6 h compared with the control (0 h), no significant differences were observed among them. As the fumigation time extended, the soluble protein content showed an increasing trend, with the variations ranging from 2.32 to 4.65 mg g^−1^ fw. The soluble protein contents did not show a significant difference between the recovery group and the control group (adjusted *p* > 0.05).

In *C. putoensis*, the soluble protein content also showed an increasing trend as the fumigation time prolonged. The variations ranged from 2.61 to 3.27 mg g^−1^ fw. Compared with the control group, the recovery group exhibited a lower soluble protein content, although no significant difference was observed between them.

As shown in Fig. 4, the maximum difference in the soluble protein content of *C. betulus* was 2.33 mg g^−1^ fw, which was greatly larger than that of *C. putoensis* (0.66 mg g^−1^ fw). Particularly, *C. betulus* exhibited a rapid increase in the soluble protein content from 12 h of fumigation, which indicates that *C. betulus* increased protein synthesis when encountered with NO_2_ stress, whereas *C. putoensis* showed only weak resistance against the stress.

### Changes in NR

**Figure 5:**
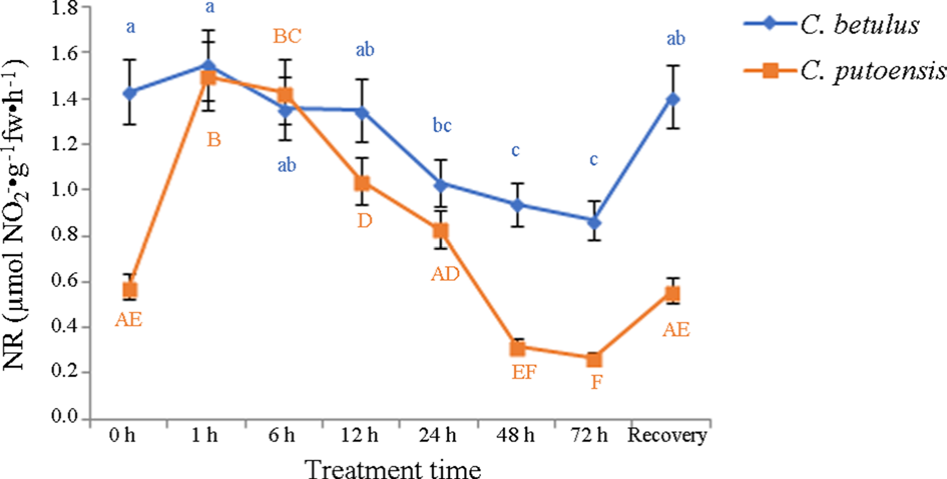
Changes in the NR activity of *C. betulus* and *C. putoensis* under NO_2_ stress at different time points and after self recovery. Letters or letter combinations containing the same letter indicate no significant difference between the corresponding NO_2_ exposure time points in the same plant species according to ANOVA or nonparametric Kruskal–Wallis ANOVA followed by Bonferroni tests. Capital letters for *C. putoensis* and lower letters for *C. betulus.*

At 0 h of NO_2_ treatment, the NR activity of *C. betulus* was 1.43 ± 0.14 µmol NO_2_^−^·g^−1^fw·h^−1^. With the prolongation of NO_2_ exposure, the NR activity of *C. betulus* exhibited a gradual increase followed by a gradual decrease, and a significant difference (adjusted *p* < 0.05) was observed from 24 h. After 30-d recovery, the NR activity returned to a normal level (adjusted *p* > 0.05). In *C. putoensis*, the NR activity of the control group was 0.58 ± 0.06 µmol NO_2_^−^·g^−1^fw·h^−1^. As the NO_2_ exposure time prolonged, NR activity exhibited a rapid increase (adjusted *p* < 0.05) followed by a fast decrease. After 30-d recovery, the index returned to a normal level (adjusted *p* > 0.05). The results were shown in Fig. 5.

### Changes in NO_3_^−^N

**Figure 6:**
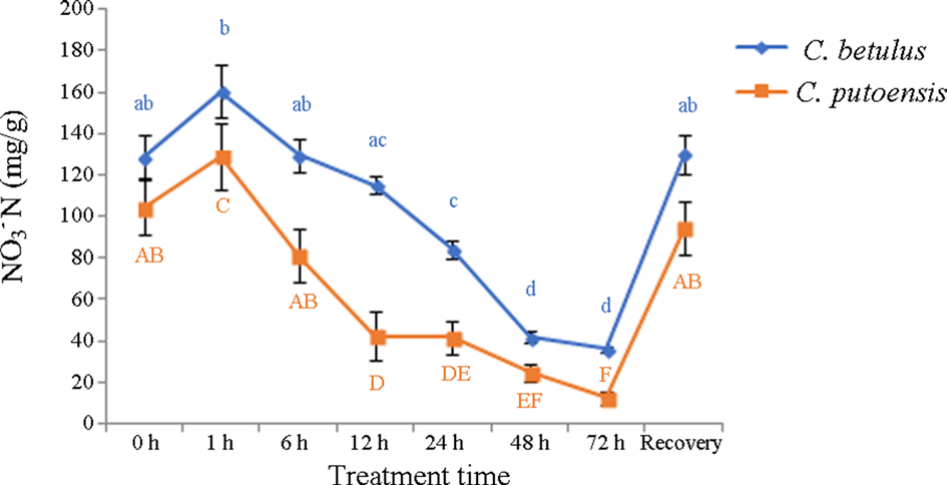
Changes in the NO_3_^−^N content of *C. betulus* and *C. putoensis* under NO_2_ stress at different time points and after self recovery. Letters or letter combinations containing the same letter indicate no significant difference between the corresponding NO_2_ exposure time points in the same plant species according to ANOVA or nonparametric Kruskal–Wallis ANOVA followed by Bonferroni tests. Capital letters for *C. putoensis* and lower letters for *C. betulus.*

As the NO_2_ treatment time extended, the NO_3_^−^N content of *C. betulus* exhibited an increase followed by a gradual decrease, and a significant difference (adjusted *p* < 0.05) was observed from 24 h. After 30-d recovery, the gradual returned to a normal level (adjusted *p* > 0.05). In *C. putoensis*, the NO_3_^−^N content also exhibited an increase followed by a decrease after NO_2_ exposure. However, a significant difference was observed from 12 h. After 30-d recovery, the index returned to a normal level (adjusted *p* > 0.05). The results were shown in Fig. 6.

### Changes in mineral elements

The changes in the mineral elements of *C. betulus* and *C. putoensi* under NO_2_ stress and after self recovery are summarized in Table 2.

**Table 2:** Changes in the mineral element contents of *C. betulus* and *C. putoensis* under NO_2_ stress and after self recovery.

Mineral element	Treatment							
	CK	1 h	6 h	12 h	24 h5	48 h	72 h	Recovery
**N (g/kg** **dw)**								
*C. betulus*	1.4 ± 0.13^a^	1.68 ± 0.17^ab^	1.12 ± 0.21^ac^	1.12 ± 0.11^ac^	1.12 ± 0.11^ac^	1.12 ± 0.11^ac^	0.84 ± 0.08^c^	1.53 ± 0.15^ad^
*C. putoensis*	1.12 ± 0.11^a^	1.68 ± 0.15^b^	1.12 ± 0.11^a^	1.12 ± 0.11^a^	0.98 ± 0.32^a^	0.84 ± 0.08^a^	0.46 ± 0.05^c^	1.12 ± 0.11^a^
**P (ppm dw)**								
*C. betulus*	0.72 ± 0.06^a^	2.14 ± 0.21^b^	2.76 ± 0.26^b^	2.2 ± 0.21^b^	2.5 ± 0.24^b^	4.69 ± 0.51^c^	4.35 ± 0.44^c^	0.93 ± 0.08^a^
*C. putoensis*	1.85 ± 0.20^a^	4.78 ± 0.45^b^	4.35 ± 0.44^b^	2.01 ± 0.20^ac^	3.11 ± 0.29^d^	2.95 ± 0.30^cd^	2.41 ± 0.26^ad^	2.03 ± 0.20^a^
**K (µg L**^−**1**^ **dw)**								
*C. betulus*	20.9 ± 2.07^ab^	18.6 ± 1.91^abc^	16.1 ± 1.59^bcd^	15.2 ± 1.49^cde^	14.5 ± 1.45^cde^	12.1 ± 1.21^de^	11.4 ± 1.14^de^	21.6 ± 2.16^a^
*C. putoensis*	30.2 ± 3.03^d^	14.2 ± 1.38^bc^	16.5 ± 1.65^bc^	13.2 ± 1.29^ac^	9.8 ± 0.98^a^	11.3 ± 1.13^a^	10.8 ± 1.08^a^	18.9 ± 1.89^b^
**Ca (µg L**^**−1**^ **dw)**								
*C. betulus*	232 ± 24.02^b^	243 ± 23.90^b^	216 ± 31.00^b^	198 ± 20.01^b^	223 ± 12.00^b^	188 ± 18.80^b^	84 ± 8.38^a^	230 ± 13.00^b^
*C. putoensis*	237 ± 23.80^e^	149 ± 15.10^a^	172 ± 17.20^ad^	205 ± 21.10^cde^	168 ± 17.01^ac^	159 ± 16.01^ab^	137 ± 14.02^a^	240 ± 23.89^e^
**Mg (µg L**^−**1**^ **dw)**								
*C. betulus*	23.2 ± 2.29^ac^	27.3 ± 1.70^af^	26.5 ± 3.60^ae^	28 ± 4.80^ag^	26.1 ± 1.60^ad^	31.3 ± 3.13^bdefg^	21.4 ± 2.14^a^	30.5 ± 3.05^cdefg^
*C. putoensis*	26.8 ± 2.71^ cd^	32.2 ± 3.22^d^	22.4 ± 2.24^bc^	19.5 ± 1.95^ac^	19.4 ± 3.90^ab^	12.2 ± 1.22^a^	22.6 ± 2.26^bc^	30.7 ± 3.07^d^
**Zn (µg L**^−**1**^ **dw)**								
*C. betulus*	7.3 ± 0.73^a^	10.2 ± 0.45^bd^	10.5 ± 1.50^ cd^	10.6 ± 1.06^ cd^	9 ± 0.90^ad^	7.1 ± 0.69^a^	7.4 ± 0.72^ab^	11.6 ± 1.09^ cd^
*C. putoensis*	7.1 ± 0.68^bc^	6.3 ± 0.36^ac^	4.5 ± 0.75^a^	7.2 ± 0.72^bc^	5.7 ± 0.61^ab^	11.2 ± 1.12^d^	7.9 ± 0.79^bc^	10.3 ± 1.03^d^
**Mn (µg L**^**−1**^ **dw)**								
*C. betulus*	107.6 ± 12.70^d^	11.2 ± 5.60^a^	59.3 ± 9.40^bc^	50.6 ± 5.06^b^	78.1 ± 7.81^cef^	67.7 ± 3.70^be^	49.9 ± 3.70^b^	90.2 ± 9.02^df^
*C. putoensis*	87.3 ± 8.75^c^	85.5 ± 12.70^c^	62.3 ± 6.18^b^	47.1 ± 4.69^b^	21.4 ± 2.09^a^	11.8 ± 2.60^a^	9.4 ± 1.40^a^	80.3 ± 7.98^c^

#### Macroelements

(1) N. At 1 h of NO_2_ stress, the total nitrogen content of *C. betulus* increased slightly to 1.68 ± 0.17 g/kg; this value was higher than that of control (1.4 ± 0.13 g/kg), but no significant difference was observed (adjusted *p* > 0.05). With the prolongation of the stress, the content decreased, with the variations ranging from 0.84 to 1.68 g/kg and the maximum difference of 0.84 g/kg. The recovery group did not show a significant difference compared with the control group (1.53 ± 0.15 vs. 1.4 ± 0.13; adjusted *p* = 1.00).

Overall, the changes in the total nitrogen content of *C. putoensis* showed a similar trend with those of *C. betulus.* At 1 h of NO_2_ stress, the total nitrogen content of *C. putoensis* significantly increased compared with that of the control (1.68 ± 0.15 g/kg vs. 1.12 ± 0.11 g/kg; adjusted *p* < 0.001). With the prolongation of NO_2_ fumigation, the content gradually decreased, with the variations ranging from 0.46 to 1.68 g/kg and the maximum difference of 1.22 g/kg. No significant difference was observed between the recovery group and the control group (adjusted *p* = 1.00). Although both species showed noticeable changes in the total nitrogen content compared with their corresponding control, the variation amplitude of *C. putoensis* was much greater than that of *C. betulus* (1.22 g/kg vs. 0.84 g/kg).

(2) P. As the NO_2_ stress prolonged, the P content of *C. betulus* increased, showing significant differences compared with the control. The variations ranged from 0.72 to 4.69 ppm dw, with the maximum difference of 3.97 ppm dw. No significant difference was observed between the recovery group and the control group (0.72 ± 0.06 vs. 0.93 ± 0.08; adjusted *p* = 1.00). Compared with the control, the P content of *C. putoensis* undergoing NO_2_ stress showed an increase followed by a decrease. The variations ranged from 1.85 to 4.78 ppm dw with the maximum difference of 2.93 ppm dw.

(3) K. With the prolongation of NO_2_ exposure, the K content of *C. betulus* gradually decreased, and a significant difference was observed from 12 h. The variations in the K content ranged from 11.4 to 21.6 µg L^−1^ dw, with maximum difference of 10.2 µg L^−1^ dw. The K content of *C. putoensis* showed a similar trend to that of *C. betulus*. The variations in the K content ranged from 9.8 to 30.2 µg L^−1^ dw. Compared with *C. betulus*, *C. putoensis* exhibited a relatively greater amplitude of the variations in the K content (10.2 µg L^−1^ vs. 20.4 µg L^−1^ dw). In both species, the recovery groups did not show a significant difference compared with the control (adjusted *p* > 0.05).

(4) Ca. With the prolongation of NO_2_ exposure, the Ca content of *C. betulus* exhibited an increase followed by a gradual decrease, and the variations ranged from 84 to 243 µg L^−1^ dw. A significant difference was observed at 72 h of NO_2_ exposure. In *C. putoensis*, significant differences in the Ca content were observed during NO_2_ exposure, except at 12 h. In both species, the Ca content of the recovery group did not show a significant difference compared with the control group. The variation amplitude of the Ca content of *C. betulus* (159 µg L^−1^ dw) was noticeably greater than that of *C. putoensis* (68 µg L^−1^ dw).

(5) Mg. As the NO_2_ stress prolonged, the Mg content of *C. betulus* did not show a significant drop, except at 48 h. The variations ranged from 21.4 to 31.3 µg L^−1^ dw. In *C. putoensis*, the variations ranged from 12.2 to 32.2 µg L^−1^ dw. In both species, the Ca content of the recovery group did not show a significant difference compared with the control group. The variation amplitude of the Ca content of *C. betulus* (9.9 µg L^−1^ dw) was remarkably smaller than that of *C. putoensis* (20 µg L^−1^ dw).

#### Microelements

(1) Zn. With the prolongation of NO_2_ exposure, the Zn content of *C. betulus* exhibited an increase followed by a gradual decrease. Compared with the control, significant differences were observed at 1, 6, and 12 h. The variations anged from 7.1 to 10.6 µg L^−1^ dw. In *C. putoensis*, significant differences in the Zn content were observed at 6 h and 48 h, and the variations ranged from 5.7 to 11.2 µg L^−1^ dw. The variation amplitude of the Zn content of *C. betulus* (3.5 µg L^−1^ dw) was smaller than that of *C. putoensis* (5.5 µg L^−1^ dw). In each species, the Zn content of the recovery group showed a significant difference compared with the control group.

(2) Mn. At 1 h of NO_2_ fumigation, a sharp drop was observed, compared with the control. Afterwards, the Mn content of *C. betulus* exhibited a general increase followed by a gradual decrease. However, at any time point during NO_2_ exposure, a significant lower Mn content was observed when compared to the control. The variations of the Mn content ranged from 11.2 to 78.1 µg L^−1^ dw. In *C. putoensis*, the Mn content during NO_2_ exposure significantly decreased compared with control, and the variations ranged from 9.4 to 85.5 µg L^−1^ dw. The variation amplitude of the Mn content of *C. betulus* (66.9 µg L^−1^ dw) was slightly smaller than that of *C. putoensis* (76.1 µg L^−1^ dw). In each species, the Mn content of the recovery group did not show a significant difference compared with the control group.

### Correlation analysis

The correlations between the investigated indices and NO_2_ exposure time were analyzed using the Pearson’s method (Table 3). POD and soluble protein had a strong positive correlation with NO_2_ exposure time (correlation coefficient: 0.891 and 0.799, respectively), and NR, NO_3_^−^N, N, K, and Ca had a strong negative correlation with NO_2_ exposure time (correlation coefficient: -0.691, -0.805, -0.744, -0.606 and -0.696, respectively). MDA and the Zn content were not correlated with the exposure time.

**Table 3:** Correlations of the investigated indices with NO_2_ exposure time.

Index	Correlation coefficient	P value
MDA	0.153	0.334
POD	0.891	< 0.001
Soluble protein	0.799	< 0.001
NR	− 0.691	< 0.001
NO_3_^−^N	− 0.805	< 0.001
N	− 0.744	< 0.001
P	0.326	0.035
K	− 0.606	< 0.001
Ca	− 0.696	< 0.001
Mg	− 0.323	0.037
Zn	0.062	0.697
Mn	− 0.464	0.002

MDA, malondialdehyde; POD, peroxidase; NR, nitrate reductase; NO_3_^−^N, nitrate nitrogen.

## Discussion

In this study, the physiological and biochemical responses of *C. betulus* and *C. putoensis* under NO_2_ stress, as well as their self recovery after stress removal, were investigated. The resistance and metabolic capabilities of the plant species in NO_2_ adversity were then compared. The results showed that the influence of different NO_2_ stress durations on the physiological and biochemical responses of the plants differed, which exhibited different levels.

Under NO_2_ stress, the MDA content in *C. putoensis* increased with the prolongation of NO_2_ exposure, and a significant increase was observed from 24 h, compared with the control. Yuan et al.^23^ investigated the effect of NaCl stress on the physiological and biochemical characteristics of *Pistacia vera* and found that the MDA level in the plant noticeably increased with the stress. Our result was basically consistent with that reported in the literature. MDA is a cytotoxic substance. After plant organs are damaged by NO_2_ pollution, accumulated MDA injures leaf membranes and cells, leading to membrane lipoperoxidation^23^. Tiwari et al.^24^ investigated the impact of ambient air pollution on *Dacus carota*var and found that the lipoperoxidation increased in plants under oxidative stress caused by SO_2_, NO_2_ and O_3_. Wang et al.^25^ found that 16.0 μl/L NO_2_ caused necrotic lesions on tobacco seedling leaves, which led to cell membrane damage. However, in this study, a significant difference in MDA was not observed in *C. betulus*. Furthermore, the variation amplitude of *C. betulus* was smaller than that of *C. putoensis*. These results suggest that *C. betulus* has a higher resistance against NO_2_ stress than *C. putoensis*. After 30-d self recovery, the MDA content of both plant species decreased, which did not show a significant difference compared with that of the control. This finding suggests that NO_2_ exposure-caused damage can be recovered via self metabolisms of the plants after some time of normal cultivation at room temperature. However, our study did not show a correlation with NO_2_ exposure time. Presumably, this outcome was caused by a small sample size in this study, and therefore, studies with a larger sample size remain to be conducted in the future.

POD serves as an essential protective enzyme for cells to defense reactive oxygen damage, which reflects the resistance of plants against adversity^26^. In this study, the POD activity of both *C. betulus* and *C. putoensis* increased with the prolongation of NO_2_ fumigation. In Ma’s study^18^, 5 different genotypes of *Arabidopsis thaliana* were ventilated with NO_2_ at a concentration of 6 ppm for 7 days, with 3 h per day; NO_2_ stress caused an increase in POD activity. Our results were in basic consistency with that reported by Ma. However, in Ma’s study, he did not observe the dynamic change in POD with the prolongation with NO_2_ fumigation. Compared with the control, *C. betulus* showed a significant difference in POD activity from 48 h, which reached the peak at 72 h. In *C. putoensis*, POD activity significantly increased from 24 h of NO_2_ fumigation. These findings indicate that the appearance of damage reactions in *C. putoensis* was earlier than that of *C. betulus*. Under long-term NO_2_ stress, active oxygen is produced in organisms to lead to accumulation. When the accumulated active oxygen exceeds the capacity of the active oxygen clearance system of the plant, oxidative damage will appear. In this condition, plants will present with noticeable damage reactions^27^. In this study, *C. betulus* did not show a significant increase in POD activity within 48 h of NO_2_ fumigation, which indicates that the produced active oxygen during this period was still within the range of the amount the active oxygen clearance system could remove. In contrast, the POD activity of *C. putoensis* significantly increased from 24 h of NO_2_ fumigation, which indicates a weaker active oxygen clearance capacity. After 30-d self recovery, the POD activity of both species decreased, which did not show a significant difference compared with the control. Presumably, plants might recover from NO_2_ caused damage through metabolism after some time of self recovery^28^.

In adverse environment, plants increase the synthesis of soluble protein to directly participate in substance production during the process of adaptation^29^. In this study, although the soluble protein contents of both *C. betulus* and *C. putoensis* increased with the prolongation of NO_2_ stress, the increase in the former was more steady. Zhang et al.^30^ investigated the physiological and biochemical changes of *Euonymus japonicus* and *Chaenomeles speciosa* under SO_2_ stress; although the soluble protein content was increased in both plants treated with SO_2_, the increase ratio of *Euonymus japonicus* was greater than that of *Chaenomeles speciosa*. Their results and the results of this study indicate that the levels of anti-pollution basic substances in resistant plants are higher than those of sensitive plants. In this study, the increase amplitude of *C. betulus* under NO_2_ stress was greater than that of *C. putoensis*, which suggests that *C. betulus* possesses more satisfactory adaptability to NO_2_ exposure than *C. putoensis*. After 30-d self recovery, the soluble protein level of both plant species decreased. However, the level of *C. betulus* remained higher than that of *C. putoensis*. Soluble protein serves as the main form of nitrogen in plants, whose content is closely associated with the metabolism and aging of the plants; in the meantime, it is also greatly associated with the osmotic pressure and anti-dehydrate maintained by the plant^28^. Based on the literature^28^, the results of this study seem to suggest that under NO_2_ stress, the metabolic activity and protein synthesis in *C. betulus* might increase to maintain normal osmotic pressure in vivo.

In this study, with the prolongation of NO_2_ exposure, the NO_3_^−^N content in both species increased followed by a decrease. After 30 days of recovery, the content increased close to a normal level. In addition, as the NO_2_ exposure time prolonged, the NR activity of both species showed an increase followed by a decrease. These findings were basically consistent with the results reported by Teng et al.^31^. However, the variation trend of NR activity in *C. betulus* was less noticeable than that of *C. putoensis*, which indicates that the synthesis of NR in *C. betulus* was more stable under NO_2_ stress, and therefore, *C. betulus* possesses higher adaptability to external adverse environment compared with *C. putoensis.*

In this study, the changes in biochemical elements in *C. betulus* and *C. putoensis* were also determined. Under NO_2_ stress, the N content of both species showed an increase (at 1 h) followed by a decrease, compared with the control. Since dissolution of NO_2_ in the cell sap may produce nitrate and nitrite ions, which can be assimilated into organic nitrogenous compounds by the plant, NO_2_ may serve as a source of N^32,33^. Our finding indicates that short-term NO_2_ stress benefits the supply of N sources for the plants. However, long-time stress is harmful to the synthesis of N compounds. Presumably, NO_2_ stress has an impact on stomatal aperture. At the early period of stress, the stomatal aperture is normal and the N source supply is sufficient. As the stress prolongs, plants decrease or even close the stomatal aperture to resist the external stress and maintain normal metabolisms. In the meantime, the chlorophyll content decreases, the structure of chloroplasts is damaged, and photosynthesis is blocked^22^. In this study, the variation amplitude of the N content of *C. betulus* (0.84 g/kg) was noticeably smaller than that of *C. putoensis* (1.22 g/kg), which suggests that *C. putoensis* has larger metabolic fluctuations when exposed to high-concentration NO_2_, and therefore, is more sensitive to the changes in external environment. P is a component of nucleric acids, proteins and phosphatides; it plays an important role in cell division and genetic information transmission, as well as in carbohydrate and energy metabolism^34^. At 1-h NO_2_ exposure, both plant species showed a significant increase in the leaf P content compared with the control and recovery groups, which suggests that a certain amount of NO_2_ benefits P synthesis in the plants. However, with the stress time extended, the P content of *C. betulus* showed a gradually increasing trend, whereas that of *C. putoensis* exhibited a gradually decreasing trend (except for 12 h). This finding suggests that the effect of NO_2_ stress on P synthesis may exhibit differences according to species. K promotes the transmission of carbohydrates to storage organs and enhances the hydration of protoplasms; it strengthens cellular water holding capacity as well as drought resistance of the plant^35^. When K is sufficient, protein production increases while soluble N decreases. In this study, the K content of both *C. betulus* and *C. putoensis* under NO_2_ stress significantly decreased, compared with the control and recovery group. Presumably, to resist the stress, the protein synthesis in the plants increased, which increased K consumption, leading to a decrease in the K content. Ca is an essential mineral nutrient element for the synthesis of calcium pectinates in the middle lamella of the cell wall. It maintains the structure and functions of the cell wall and cell membrane, and serves as the second messenger for the intra- and extracellular information transfer. Furthermore, it participates in the composition of chromosome structure and maintains its stability^23^. Mg functions as the activator of a variety of enzymes. It plays an important role in the process of phosphoric acid and protein metabolism, and promotes the phosphate absorption of the plant. Therefore, plants that require much P also require much Mg. In this study, the variation amplitude of the Mg content in *C. putoensis* (20 µg L^−1^ dw) under NO_2_ stress was much greater than that of *C. betulus* (9.9 µg L^−1^ dw), which was consistent with its greater variations in the P content under NO_2_ stress in this study. Mn promotes amyloysis and saccharide transfer. Zn functions as the activator of some enzymes, which is necessary for the synthesis of tryptophan, the precursor of somatotropin; it, combined with Ca, maintains the stability and integrity of cell membranes^36^. In this study, *C. putoensis* showed a greater variation amplitude in both Mn and Zn than *C. betulus* (76.1 µg L^−1^ dw vs. 66.9 µg L^−1^ dw; 5.5 µg L^−1^ dw vs. 3.5 µg L^−1^ dw), which indicates that the contents of these microelements in *C. putoensis* vary greatly under NO_2_ stress.

It is noteworthy that in this study, the included study objects were all seedlings. In the same species, developmental age is a strong determinant of stress responses in plants and age may determine stress susceptibility^37,38^. Therefore, the selection of seedlings for the experiments in this study might cause biases to the results finally obtained in this study.

To draw a conclusion, short-term high-contraction NO_2_ stress has significant effect on the physiological and biochemical responses of *C. betulus* and *C. putoensis*. *C. betulus* and *C. putoensis* exhibited noticeable differences in resistance against the stress. According to the analysis based on lipoperoxidation and antioxidase activity, showed that the emergency reactions of *C. betulus* are more stable than those of *C. putoensis*, and therefore, *C. betulus* has stronger resistance against NO_2_ stress. To assess the resistance of plants against NO_2_ stress, the activities of membrane lipid oxygenase and antioxidase can be used as priority indices. *C. putoensis* is more sensitive to NO_2_ stress, whereas *C. betulus* possesses more powerful ionic balance maintaining capacity, which may be one of the reasons for more satisfactory adaptability of *C. betulus* when exposed to high-concentration NO_2_. However, for both *C. betulus* and *C. putoensis*, the damage caused by high-concentration NO_2_ is reversible. Therefore, these two precious species can both be applied in urban landscaping.

## Materials and methods

### Fumigation device

**Figure 7:**
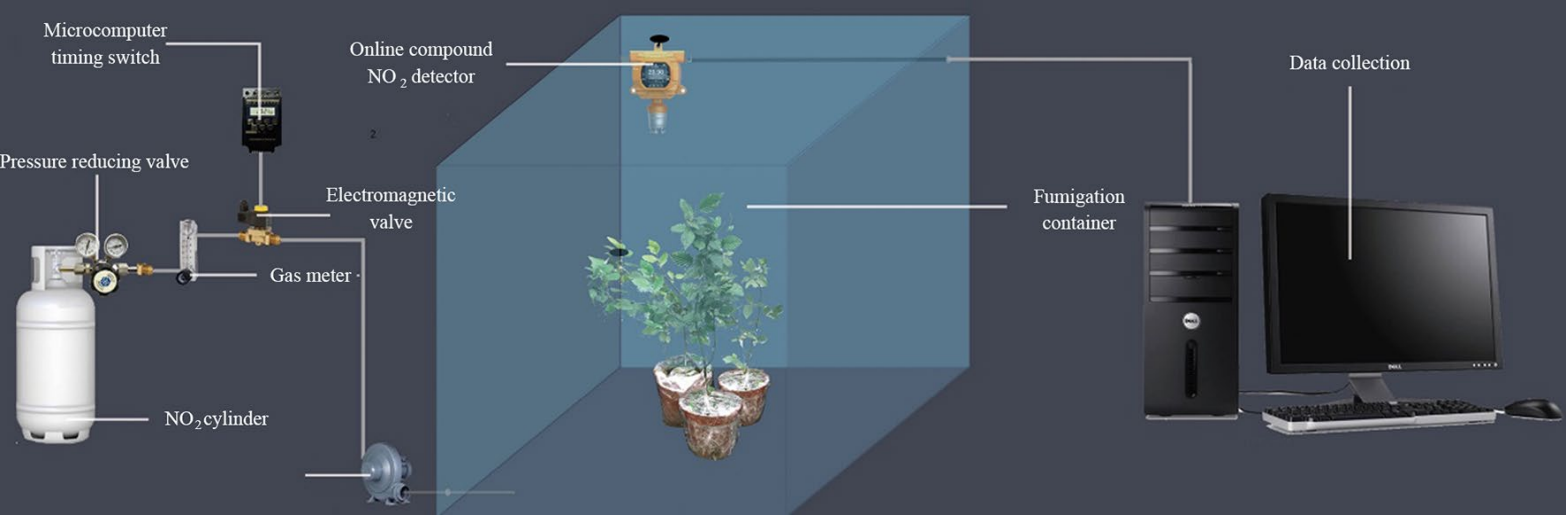
The fumigation test device for timing regulation and recording of the NO_2_ concentration (drawn with Adobe Photoshop CC2019; https://download.zol.com.cn/tuiguang/detail/46/456409.shtml?qw =).

Fumigation was performed with a self-designed patented real-time NO_2_ concentration monitoring device (patent no., ZL 2017 2 0085636X; Fig. 7). The gas outlet of the NO_2_ gas cylinder was connected to an electromagnetic valve (with a pressure reducing valve) and then a microcomputer switch timing system. An NO_2_ sensor was installed in the fumigation container to monitor the gas concentration, and the other end of the sensor was connected to the air inlet of an NO_2_ meter. The other end of the NO_2_ meter was connected to the computer terminal via an RS-485 interface. Real-time NO_2_ concentration was recorded by NO_2_ monitoring software installed in the computer. This device can control the gas volume entering the room precisely, and is convenient to manipulate^20,22^.

### Experimental materials

The experimental materials were 1-year seedlings of *Carpinus betulus* from Hungray and of *Carpinus putoensis* from Mount Putuo, China. Their height ranged from 30 to 40 cm with a crown width of 20–30 cm. The experiment was performed at the garden experiment center of Nanjing Forestry University (118.82°E, 32.08°N). Healthy seedlings that grew in uniformity without plant or pest disease were selected in April, 2017. They were cultivated in mixed soil, which had a peat soil-vermiculite-pearlite ratio of 1:1:1. The seedlings were planted in pots of 30 cm (upper diameter) × 20 cm (lower diameter) × 15 cm (height), with two in each pot. The pot contained a drainage hole at the bottom and was placed on a tray. The plants were allowed to grow naturally in uniform cultivation conditions (temperature, 25–28 °C; relative humidity, 60–70%; illumination, 26–29 klx; atmospheric pressure, 99.3–99.5 kPa) and managed regularly. During cultivation, they were watered 2–3 times per week, and approximately 1 L of Hoagland nutrient solutions was applied every two weeks. At 2 months, the NO_2_ stress experiment was performed.

### Fumigation experiment

To create NO2 stress, most studies used a concentration between 1.0 mg/m^3^ and 18.8 mg/m^3^^19,29,39–42^: 1.0–8.0 mg/m^3^ belongs to a low-stress concentration, at which long-time fumigation can be performed, such as 30 d and 60 d, whereas a concentration above 8.0 mg/m^3^ belongs to a high-stress concentration, at which plants are primarily subjected to short-time fumigation, such as 14 h and 48 h. Based on literature^19,29,41^ as well as a pilot experiment conducted the current team, the NO_2_ concentration was determined at 12.0 mg/m^3^ in this study, 60 times the national concentration threshold of China for NO_2_ pollution (0.2 mg/m^3^ for 24 h) and the longest fumigation time was set at 72 h (according to the pilot study of the current team, at such a concentration within the set longest fumigation time, plants presented with damage symptoms and emergency reactions but without deaths).

The plants were divided according to the fumigation durations of 0 (control), 1, 6, 12, 24, 48, and 72 h, with 10 plants in each group. The NO_2_ concentration was monitored by the gas meter every 1 min, and the set concentration was realized with a gas measuring flowmeter. The pots and soil were wrapped with freshness-keeping plastic films to exclude the potential influence of soil and rhizospheric microorganisms on the outcomes. The conditions for fumigation included an environmental temperature of 25–28 °C, relative humidity of 60–70%, an illumination intensity of 26–29 klx and an atmospheric pressure of 99.3–99.5 kPa. After fumigation, the plants were moved out of the fumigation room and then cultivated without NO_2_ application at room temperature for 30 d. The growth conditions were the same as were provided for the NO_2_ treatment groups.

In each treatment group, the fourth leaves from the apex of the plants were collected, whose status ranged from being healthy to being severely NO_2_ damaged. All the experiments described in the following sections were repeated thrice.

### Index determination

A number of physiological and biochemical indices are associated with NO_2_ resistance capacity of plants. In this study, MDA, = POD, soluble protein, NR and NO_3_^−^N were determined to investigate the differences between *C. betulus* and *C. putoensis* under NO_2_ stress.

#### MDA

MDA is an important product of the membrane lipid peroxidation of plants. It is negatively correlated with the severity of pollution stress, and its content reflects the severity of the adversity the plant is exposed to^23^.

MDA was measured using penthiobarbital assays. Briefly, fresh leaves at approximately 0.2 g were placed into a pre-chilled mortar and then ground with 0.2 g of quartz sands. A total of 6-mL 0.05 mol/L phosphate buffer (in three applications, including the one for mortar rinsing) was added. The obtained homogenate was stored in a 10-mL centrifuge tube at 4 °C. The sample was centrifuged at 9000 r/min for 20 min, and the obtained supernate was the crude extract of MDA. The extract was poured into a 10-mL centrifuge tube and stored at 2 °C for later use. The sample was mixed with 1 mL of 10% TCA and 1 mL of 0.67% TBA, boiled, and then immediately chilled for 15 min. Centrifugation was performed at 1800 r/min for 10 min. Then, the absorbance values at 535 nm and 600 nm were measured. The standard MDA solution was used to draw the working curve, and the MDA content in the sample was calculated^43^.

#### POD activity

POD is subject to a class I oxidation reduction enzyme that acts as a catalyst in a variety of biological processes, and it is an essential protective enzyme for cells to defense reactive oxygen damage. In adversity, POD is activated^44,45^ and exhibits resistance against adverse oxidation stress^26,46^.

POD activity was measured using guaiacol colorimetry^47^. Fresh leaves at approximately 0.2 g were placed into a pre-chilled mortar and then ground with 0.2 g of quartz sands. A total of 6 mL of 0.05 mol/L phosphate buffer (in three applications, including the one for mortar rinsing) was added. The obtained homogenate was poured into a 10-mL centrifuge tube and stored at 4 °C. The sample was centrifuged at 9000 r/min for 20 min, and the obtained supernate was the crude extract of POD. The reaction system for enzymatic activity measurement contained 2.9 mL of 0.05 mol/L phosphate buffer, 1.0 mL of 2% H_2_O_2_, 1.0 mL of 0.05 mol/L guaiacol and 0.1 mL of enzymatic solution. Enzymatic solution that was boiled for 5 min was used as the control. After enzymatic solution application, the system was immediately subjected to incubation at 37** °C** for 15 min followed by an ice bath. Trichloroacetic acid (20%) at 2.0 mL was added to terminate the reaction. Filtering and appropriate dilution were then performed. The absorbance at a wavelength of 470 nm was measured^47^.

#### Soluble protein

Soluble protein content is another important index in research on plant resistance. The soluble protein content was determined as follows^28^. Fresh leaves at approximately 0.2 g were placed into a pre-chilled mortar and then ground with 0.2 g of quartz sands. Then, 2 mL of distilled water was added. The obtained homogenate was poured into a 10-mL centrifuge tube and then kept at room temperature for 0.5–1 h. The sample was centrifuged at 4000 r/min for 20 min, and the obtained supernate was transferred into a 10-mL volumetric flask. Distilled water was added to the metered volume, and the extract to be tested was obtained. Two 10-mL plug test tubes were used, and 0.1 of the extract was applied into each of them. Coomassie brilliant blue protein reagent at 5 mL was added for intensive mixing. The mixture was allowed to stand for 2 min. With extract-free solution as the control, colorimetry was performed with a cuvette with an optical path of 1 cm at a wavelength of 595 nm, and the optical density (OD) value was determined. The soluble protein content in the sample was determined according to the standard curve^28^.

#### NR

Fresh leaves at approximately 0.2 g were cut up and placed in a deep freezer for 30 min. The samples were subjected to an ice bath and then ground with a small amount of quartz sands and 4 ml of extracting buffer. The homogenate was centrifuged at 4000 r/min at 4 °C for 15 min. The obtained raw enzyme extract (supernatant) at 0.4 ml was transferred into a 10-ml tube. Approximately 1.2 ml of 0.1 mol/L KNO_3_ phosphate buffer and 0.4 ml of NADH solution was added. The solution was incubated in 25 °C water for 30 min. For the control, NADH solution was replaced with 0.4 ml of 0.1 mol/L phosphate buffer (pH7.5). After temperature holding, 1 ml of sulfanilamide solution was immediately added to terminate reactions. Naphthylvinylamine solution at 1 ml was added for coloration for 15 min. Centrifugation at 4000 r/min was performed for 5 min. The obtained supernatant was subjected to colorimetric determination at 540 nm. The total content of NR in the reaction system was calculated based on regression equation^48^.

#### NO_3_^−^N

Fresh leaves were cut up and well mixed, and 0.2 g was applied into a 10 ml of deionized water. The tube was beaded and then placed in boiling water for 30-min extraction. Then, the sample was chilled. The extracted liquid was filtered into a 25-ml volumetric flask. Repeated washing was performed to remove residuals, and then the liquid was diluted to the required scale. Sample solution was reacted with 0.4 ml of 5% salicylic acid-sulfuric acid solution at room temperature for 20 min. NaOH (8%) at 9.5 ml was added. The sample solution was chilled to room temperature. Blank control was set. Absorbance was read at 410 nm. The NO_3_^−^N concentration was determined based on the standard curve or calculated with the regression equation for determination of the NO_3_^−^N (Nitrite^−^N) content in the sample^49^.

### Mineral elements

Mineral nutrients are essential for plant normal growth, which include macroelements (such as N, P and K), medium elements (such as Ca, Mg and S), and microelements (such as Fe, Mn, Cu, Zn, B, Mo and Cl). Mineral elements are closely associated with plant resistance. Both biological pathogens and adverse environment are connected to plants via nutrition supply. The nutritional level of a plant depends on the types of the mineral elements, as well as their proportions, in the plant; plants at the optimal nutritional level possess the most powerful resistance against diseases^50^.

The dried ground sample of 0.2 g was placed into a 100-mL Kjeldahl bottle and then moistened. Concentrated sulfuric acid at 5 mL was added, and the bottle was shaken gently. A twist-necked funnel was placed at the beak of the bottle. The sample was subjected to a slow heat on a digestive furnace. When the sulfuric acid was decomposed and white smoke rose, the temperature was increased gradually. When the solution appeared brownish black completely, heating was terminated. The solution was chilled slightly, and then 10 droplets of 300 g/L H_2_O_2_ were added. The bottle was shaken for complete reactions. The solution was heated to slight boiling and then kept for 10–20 min. After a slight chill, 5–10 droplets of H_2_O_2_ were added. These procedures were repeated 2–3 times till the digested solution appeared colorless or in a clear color. The solution was heated for 5–10 min to remove excess H_2_O_2_. The bottle was chilled and the funnel was rinsed with a small amount of water. The solution was metered to 100 mL with distilled water, and the filtered solution was used for mineral element content measurement. Blank tests were also performed to correct possible reagent errors.

N was determined using the kjeldah method^36^, P was determined using molybdenum-antimony-scandium colorimetry^35^, K was determined using flame photometry^51^, and Ca, Mg, Mn and Zn using the atomic absorption method^52^.

### Statistical analysis

All data were presented as the mean ± standard deviations and processed using Microsoft office Excel 2016 (http://soft.31uq.com/soft/42414.html?tab=2660412) and SPSS 24.0 (http://www.ddooo.com/softdown/77381.htm). Homogeneity of variances was determined using the Levene’s test. One-way analysis of variance (ANOVA) was performed to compare the physiological and biochemical changes in the same plant species at different time points of NO2 exposure. In case of heterogeneity, nonparametric Kruskal–Wallis one-factor analysis of variance was used. The Bonferroni method was used as the post-hoc test. Adjusted *p* < 0.05 was considered significantly different. The correlations between the investigated indices and the stress were analyzed using the Pearson’s method.
